# Morphological methods to evaluate protective agents against aminoglycoside-induced nephrotoxicity

**DOI:** 10.12861/jrip.2015.01

**Published:** 2015-03-01

**Authors:** Sandra Rodríguez Salgueiro, Lucía González Núñez

**Affiliations:** ^1^Electron Microscopy Laboratory, National Center for Scientific Research, Havana, Cuba; ^2^Pathology Department, Nephrology Institute, Havana, Cuba

**Keywords:** Aminoglycosides, Nephrotoxicity, Kidney

***Implication for health policy/practice/research/medical education*:**

Since morphological evaluations of renal tissue are imperative in the search for treatments against aminoglycoside nephrotoxicity, this kind of studies must pay attention to preparative techniques, such as the most sensible staining method. In our opinion, all the affected compartments (tubules, interstitium and glomeruli) should be analyzed and finally, the assessments should be carried out by trained personnel using unbiased methods.

Aminoglycosides are a family of antibiotics widely used in clinical practice. Their prescription is restricted by cytotoxicity and accumulation of antibiotic in renal tissues (1). Although renal damage induced by aminoglycosides is reversible, because the high regenerative capacity of tubular cells, it induces morbidity and sometimes is the cause of renal failure (2). Since aminoglycoside nephrotoxicity affects to 10-25% of patients during treatment (3), preservation of renal function during critical life-saving treatments is a problem that needs to be solved (4). Moreover, acute renal damage induced by aminoglycosides may compromise the patient’s life and may progress to chronic renal disease in survivors (5).

Aminoglycosides primarily affect epithelial cells of proximal tubules (6) and subsequently the interstitium (7) and glomeruli (8). Several studies have been performed to prevent aminoglycoside-induced renal damage in animal models (9-11). Histological evaluation of the kidneys is commonly made in these studies. Usually, qualitative descriptions of renal morphology using mainly hematoxylin and eosin (H&E) stained paraffin sections have been reported by scoring renal damage. Moreover, most of these morphological assessments are focused on tubular and interstitial effects of aminoglycosides (12-18). Some authors have also paid attention to glomerular effects (19,20).

Up to now, only few groups have accomplished more accurate tools to evaluate renal morphology. Our group has developed a semi-quantitative method which validates tubular and glomerular conditions using periodic acid Schiff (PAS) stained kidney sections. Quantification of glomerular and tubular damage is carried out by means of a program developed for .Net platform, using the integrated development environment Visual Studio 2008 and c# language, whereby the normal condition of each variable is classified as 0 and the pathological condition is considered 1. For each variable the percentage of damaged tubules or glomeruli per histological field is calculated, by means of this program. Using this method, we have evaluated some cytoprotective treatments against kanamycin nephrotoxicity in rodents (21,22).

On the other hand, Tavafi et al., have studied the volume density of proximal convoluted tubules per cortex and the glomerular volume per kidney, using stereological techniques in PAS stained kidney sections of rats treated with cytoprotective extracts and gentamicin (23,24).

Interestingly, the group of Stojiljkovic et al., has evaluated kidney histological sections using not only the conventional stains H&E and PAS, but also the Jones methenamine silver stain. They have considered the effects of some protective agents on glomerular morphometric variables (size, area, major and minor axes, perimeter, optical density and roundness of glomeruli, and also glomerular basement membrane thickness) in a model of nephrotoxicity induced by gentamicin in rats (25,26).

Since morphological evaluations of renal tissue are imperative in the search for treatments against aminoglycoside nephrotoxicity, this kind of studies must pay attention to preparative techniques, such as the most sensible staining method. In our opinion, all the affected compartments (tubules, interstitium and glomeruli) should be analyzed; and finally, the assessments should be carried out by trained personnel using unbiased methods.

## Authors’ contributions

All authors contributed to the paper equally.

## Conflict of interests

The author declared no competing interests.

## Ethical considerations

Ethical issues (including plagiarism, misconduct, data fabrication, falsification, double publication or submission, redundancy) have been completely observed by the authors.

## Funding/Support

None.
